# A Rare Case of COPA Syndrome: Multisystem Relapse and Fatal Septic Complication

**DOI:** 10.1002/rcr2.70742

**Published:** 2026-09-03

**Authors:** Flavia Castro Velasco Fernandes, Pedro Paulo Teixeira e Silva Torres, Elisângela de Paula Silveira‐Lacerda, Matheus Rabahi, Lucas Velasco Magalhaes Fernandes, Laís Rocha Lopes, Marise Amaral Rebouças Moreira, Marcelo Fouad Rabahi

**Affiliations:** ^1^ Faculdade de Medicina, Universidade Federal de Goiás Goiânia Brazil; ^2^ Hospital Israelita Albert Einstein Goiânia Goiás Brazil; ^3^ Instituto de Ciências Biológicas, Universidade Federal de Goiás Goiânia Brazil; ^4^ Lake Erie College of Osteopathic Medicine Bradenton Florida USA; ^5^ Department of Pathology Universidade Federal de Goiás Goiânia Brazil

**Keywords:** COPA syndrome; autoimmune disorder, gene mutation, interstitial lung disease

## Abstract

COPA syndrome is a rare autosomal dominant autoimmune disorder caused by COPA gene mutations, leading to immune dysregulation and multisystem involvement. We report a 45‐year‐old man with recurrent respiratory failure, haemoptysis, renal dysfunction and arthritis. Symptoms began at Age 27 with diffuse alveolar damage requiring mechanical ventilation. Imaging showed progressive interstitial lung disease and biopsy revealed follicular bronchiolitis. Immunosuppression provided temporary stabilisation. In 2024, next‐generation sequencing confirmed a likely pathogenic COPA variant. The patient later discontinued therapy and remained untreated for 1 year, returning with worsening renal dysfunction and significant proteinuria, while pulmonary findings remained stable. Cyclophosphamide was initiated for suspected renal relapse. During treatment, he developed acute diverticulitis complicated by sepsis, progressing to septic shock and multiorgan failure despite surgery and antibiotics, resulting in death. Early recognition and multidisciplinary management are essential to improve outcomes of the disease.

## Graphical abstract

A 45‐year‐old man with a history of recurrent respiratory failure, arthritis and renal dysfunction was diagnosed with COPA syndrome through genetic testing after years of progressive interstitial lung disease and immune‐mediated manifestations. He initially responded to immunosuppressive therapy but relapsed after treatment discontinuation, developing worsening renal disease that required cyclophosphamide. His course was complicated by severe infection during immunosuppression, leading to septic shock and death.

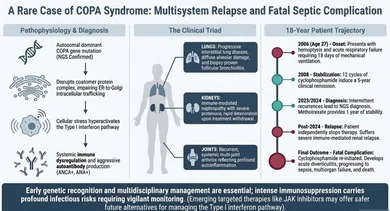

## 1 Introduction

COPA syndrome is a rare genetic autoimmune disorder that predominantly affects the lungs, kidneys and joints. It results from mutations in the COPA gene, which encodes the alpha subunit of the coatomer protein complex responsible for intracellular protein trafficking [1]. These mutations disrupt cellular homeostasis, leading to immune dysregulation characterised by autoantibody production and inflammatory responses [2]. While mutations in the COPA gene can be sporadic, the condition is often inherited in an autosomal dominant manner [1, 3]. The syndrome manifests as a combination of autoimmune and auto inflammatory features, including recurrent inflammation, haemoptysis, arthritis and nephropathy, with considerable variability in symptom severity and organ involvement [1, 3].

COPA syndrome is diagnosed by identifying heterozygous pathogenic variants in the COPA gene in patients presenting with characteristic clinical features. The classic clinical triad includes pulmonary involvement (particularly interstitial lung disease or diffuse alveolar haemorrhage), arthritis and kidney disease [1, 3].

## 2 Case Report

We describe the case of a 45‐year‐old male patient who presented in 2023 with a long history of recurrent respiratory and renal symptoms, as well as nonspecific arthralgia, dating back to 2006. At the age of 27, the patient experienced flu‐like symptoms, hemoptysis, and acute respiratory failure (ARF), necessitating orotracheal intubation (OTI) and mechanical ventilation (MV) for 18 days. Chest computed tomography (CT) at the time revealed diffuse alveolar damage (DAD) and nonspecific interstitial pneumonia (NSIP) pattern. This marked the onset of recurrent respiratory symptoms. The same year, he developed signs of renal dysfunction, followed by another episode of ARF 1 year later, which did not require OTI or MV.

At the time of the initial presentation, before the diagnosis of COPA syndrome was established, the leading clinical consideration was a pulmonary–renal syndrome, such as ANCA‐associated vasculitis and Goodpasture syndrome, among other immune‐mediated conditions. Based on this working diagnosis and the severity of organ involvement, immunosuppressive treatment with 12 cycles of cyclophosphamide was initiated in 2008. Although partial clinical stabilisation was achieved, serial chest computed tomography demonstrated progressive fibrotic interstitial lung abnormalities, including the development of upper lobe–predominant honeycombing, increasing reticulation, traction bronchiectasis/bronchiolectasis and persistent ground‐glass opacities, despite treatment. The recurrent course, progressive radiologic abnormalities the recurrent course, progressive pulmonary findings and absence of a definitive autoimmune diagnosis prompted continued investigation over subsequent years. Notably, the patient denied any family history of autoimmune diseases, interstitial lung disease, renal disease or other similar multisystemic manifestations and genetic testing was not available for the patient's family at that time.

**FIGURE 1:**
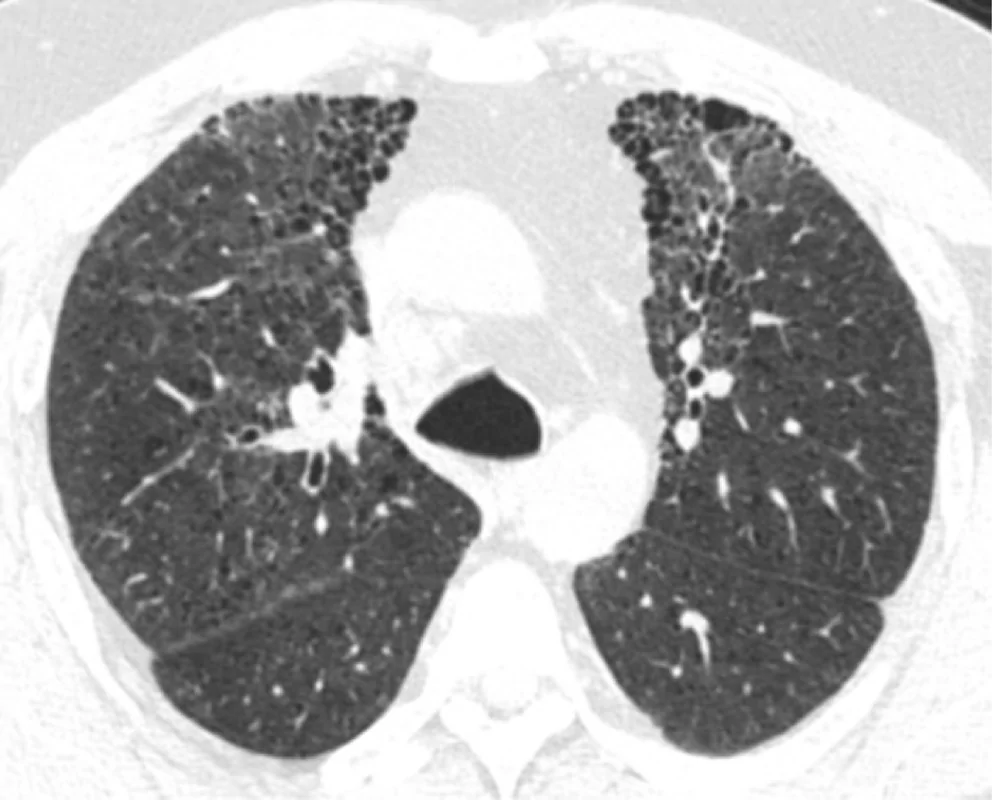
High‐resolution chest CT (lung window) demonstrating upper lobe–predominant subpleural fibrotic interstitial lung disease, characterised by bilateral honeycombing, reticulation and associated traction bronchiectasis/bronchiolectasis.

**FIGURE 2:**
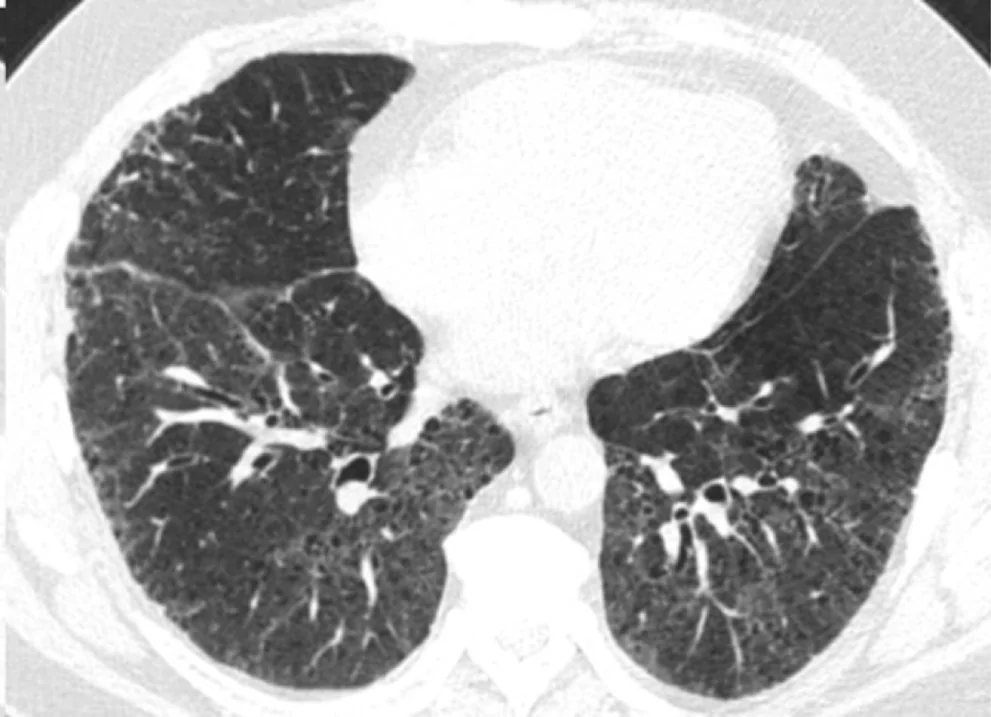
High‐resolution chest CT (lung window) demonstrating fibrotic interstitial lung disease of indeterminate pattern, characterised by bilateral mild ground‐glass opacities and reticular abnormalities associated with traction bronchiectasis.

Chest CT scans performed in 2022 demonstrated progressive bilateral fibrotic interstitial lung abnormalities. The predominant findings included upper lobe–predominant subpleural honeycombing associated with coarse reticulation and traction bronchiectasis/bronchiolectasis (Figure 1), indicating established fibrotic remodelling. Additional areas of bilateral ground‐glass opacities and fine reticular infiltrates associated with traction bronchiectasis were also present (Figure 2), suggesting superimposed active inflammatory lung injury. The coexistence of fibrotic and inflammatory abnormalities is consistent with the heterogeneous pulmonary manifestations described in COPA syndrome and reflects the combination of chronic interstitial remodelling and recurrent episodes of diffuse alveolar injury. Overall, these findings were interpreted at multidisciplinary discussion as being most consistent with fibrotic interstitial lung disease of indeterminate pattern according to current ATS/ERS criteria. A transbronchial lung biopsy was initially performed but yielded limited, nondiagnostic tissue. Because the clinical and radiologic abnormalities remained unexplained, a surgical lung biopsy was subsequently obtained to provide larger and more representative samples for histopathologic evaluation. The surgical biopsy demonstrated prominent lymphoid follicles consistent with follicular bronchiolitis, a homogeneous non‐fibrotic nonspecific interstitial pneumonia pattern with marked interstitial septal edema, areas of organising pneumonia containing intra‐alveolar fibrin consistent with an acute fibrinous and organising pneumonia pattern, fibrin thrombi within alveolar spaces and both recent and remote diffuse alveolar haemorrhage characterised by hemosiderin‐laden macrophages. No significant architectural distortion, fibroblastic foci, patchy fibrosis or honeycombing were identified. These histopathologic findings were consistent with interstitial lung disease associated with COPA syndrome, although definitive diagnosis required multidisciplinary clinicopathologic correlation.

Given the patient's pulmonary–renal syndrome, characterised by recurrent diffuse alveolar haemorrhage and proteinuria, with an initial working diagnosis that included Goodpasture syndrome and ANCA‐associated vasculitis, a renal biopsy was performed in November 2008. Histopathological examination demonstrated focal proliferative and necrotising glomerulonephritis with early epithelial crescent formation, identified in six of nine examined glomeruli. These findings supported active immune‐mediated renal involvement and, in conjunction with the subsequent genetic diagnosis, were consistent with nephropathy associated with COPA syndrome.

The results of the initial autoimmune serologic evaluation performed at the patient's first presentation in another hospital, including whether anti‐glomerular basement membrane antibody testing was performed, were unavailable for retrospective review. During reevaluation at our institution between 2021 and 2022, persistent pulmonary and renal involvement prompted a comprehensive autoimmune investigation to reassess the differential diagnosis, including connective tissue disease and ANCA‐associated vasculitis. Renal evaluation in January 2022 demonstrated impaired kidney function, with a creatinine clearance of 60 mL/min and 24‐h proteinuria of 907.5 mg/24 h. Serologic testing revealed positive antinuclear antibodies (1:320, homogeneous pattern) in December 2021 and positive p‐ANCA (1:80) in February 2022. Anti‐myeloperoxidase and anti‐proteinase 3 antibodies were negative, as were anti‐double‐stranded DNA antibodies (tested twice in February 2022), rheumatoid factor, anti‐Smith, anti‐ribonucleoprotein, anti‐Ro/SSA and anti‐La/SSB antibodies. Although anti‐glomerular basement membrane antibody results were unavailable, Goodpasture syndrome was subsequently excluded by immunofluorescence analysis of the renal biopsy, which showed no linear glomerular basement membrane immunoglobulin deposition.

In January 2024, next‐generation sequencing (NGS) identified a heterozygous COPA variant, NM_004371.4:c.698G > A (p.Arg233His), classified as likely pathogenic according to ACMG/AMP criteria (PS4, PM2, PP2, PP3 and PP4), confirming the diagnosis of COPA syndrome and explaining the patient's multisystemic manifestations. This variant has been previously reported in individuals with COPA syndrome and has been associated with pulmonary and autoimmune manifestations [4].

Because the patient received care at multiple institutions over nearly two decades, complete longitudinal pulmonary investigations, including serial pulmonary function tests, 6 min walk tests and imaging studies, were unavailable for retrospective review. The results presented below represent the available assessments performed during evaluation at our institution.

Finally, pulmonary function testing performed after treatment in August 2022 demonstrated a mixed moderate obstructive and restrictive ventilatory defect, with a forced vital capacity (FVC) of 2.10 L (53% predicted), forced expiratory volume in 1 s (FEV1) of 1.43 L (44% predicted) and an FEV1/FVC ratio of 68%, with no significant bronchodilator response. Diffusing capacity for carbon monoxide (DLCO) measured in September 2022 was markedly reduced at 42% of the predicted value. A 6 min walk test performed in December 2021 showed a baseline oxygen saturation of 96%, decreasing to 85% after walking 285 m (49% of the predicted distance), consistent with significant exercise‐induced desaturation.

After the diagnosis of COPA syndrome was established, the nephrology team initiated maintenance immunosuppressive therapy with methotrexate (10 mg/week) and prednisone (10 mg/day), which were started concurrently. The planned treatment strategy consisted of long‐term maintenance therapy with these agents together with regular clinical follow‐up to monitor pulmonary and renal disease activity. The patient achieved clinical stability and remained asymptomatic for approximately 1 year. However, he became poorly adherent to treatment and frequently self‐adjusted his medications. However, the patient frequently self‐adjusted his medications, discontinued methotrexate without medical advice because he attributed his symptoms to a viral infection, and subsequently failed to attend scheduled follow‐up visits. As a result, he remained without immunosuppressive therapy for approximately 1 year before returning with worsening renal function characterised by significant proteinuria, while pulmonary disease remained clinically and radiologically stable. Because of suspected immune‐mediated renal relapse, cyclophosphamide pulse therapy together with pulse methylprednisolone was initiated.

The patient received one cycle of cyclophosphamide before clinical deterioration occurred shortly after. Cyclophosphamide was administered at a dose of 1 g daily for 3 consecutive days, together with pulse methylprednisolone at a dose of 1 g daily for 3 consecutive days. No neutropenia was documented during treatment, and dose adjustment for renal function was not considered necessary at the time of administration. No additional immunosuppressive agents were administered during this treatment period.

During treatment, the patient developed acute diverticulitis complicated by sepsis secondary to a perforated diverticulum. There was no documented history of diverticular disease prior to this event. In November 2026, the patient developed acute perforated diverticulitis complicated by sepsis and underwent an emergency Hartmann's procedure (rectosigmoidectomy with end colostomy) for surgical source control. Detailed operative findings, including the extent of peritoneal contamination, were unavailable for retrospective review. Microbiologic culture of the abdominal cavity was not performed at the time of surgery, and therefore no microbiologic data were available for retrospective review. Despite emergency abdominal surgery and prolonged broad‐spectrum antibiotic therapy, his condition progressed to septic shock and multiorgan failure, resulting in death.

## 3 Discussion

COPA syndrome is a rare autosomal dominant disorder characterised by immune dysregulation and inflammation. It commonly presents with a triad of interstitial lung disease, arthritis and nephropathy, although phenotypic variability is significant [3]. Symptoms often begin in childhood or early adulthood, with most cases identified in patients with a family history of autoimmune conditions [1]. However, sporadic cases, like the one described here, also occur. The pathogenic mechanism of COPA syndrome involves mutations in the COPA gene, which encodes a subunit of the coatomer protein complex responsible for intracellular trafficking between the endoplasmic reticulum and Golgi apparatus [1]. Mutations disrupt protein transport, leading to cellular stress and activation of the Type I interferon pathway, which drives autoimmunity [2]. Autoantibodies, such as anti‐nuclear antibodies (ANA) and anti‐neutrophil cytoplasmic antibodies (ANCA), are frequently detected, reflecting systemic immune dysregulation [1].

This case highlights several clinically relevant aspects of COPA syndrome. First, although symptom onset occurred in early adulthood, the diagnosis was established only after a prolonged disease course spanning nearly two decades. Such diagnostic delay illustrates the challenge of recognising COPA syndrome in adult patients, particularly in the absence of a clear family history and in the setting of overlapping pulmonary, renal and autoimmune manifestations. The eventual identification of a likely pathogenic COPA variant was essential for establishing the diagnosis and integrating the patient's multisystem presentation (Table 1).

**TABLE 1:** Comparison of the present case with previously published cases of adult COPA syndrome.

Study	Age	Pulmonary features	Renal/other	Outcome	Key teaching point
Present case	45 M (onset 27)	DAH, fibrotic ILD, follicular bronchiolitis, cellular NSIP, AFOP	Crescentic GN, proteinuria, arthralgia	Fatal perforated diverticulitis with septic shock during immunosuppression	Adult sporadic presentation with diagnostic delay
Mallea et al. [5]	24 F	End‐stage ILD	Immune‐mediated kidney disease	Bilateral lung transplantation	Progression to end‐stage lung disease
Riddell et al. [6]	38 F	ILD after lung transplant	Preserved renal function, non‐nephrotic proteinuria	Progressive allograft dysfunction	Persistent immune activation after transplantation

Abbreviations: AFOP, acute fibrinous and organising pneumonia; DAH, diffuse alveolar haemorrhage; GN, glomerulonephritis; ILD, interstitial lung disease; NSIP, nonspecific interstitial pneumonia.

Distinguishing COPA syndrome from autoimmune‐associated interstitial lung disease (CTD‐ILD) can be challenging because both conditions may present with interstitial lung disease, positive autoimmune serology and multisystem involvement. However, COPA syndrome should be suspected in patients with diffuse alveolar haemorrhage, unexplained pulmonary–renal syndrome, follicular bronchiolitis, recurrent inflammatory episodes or interstitial lung disease occurring in childhood or early adulthood, particularly when accompanied by positive autoantibodies but without a definitive connective tissue disease diagnosis. Although a positive family history may raise suspicion, sporadic cases also occur. In such patients, especially those with persistent diagnostic uncertainty despite multidisciplinary evaluation, genetic testing should be considered to establish the diagnosis and guide management.

In the present case, Goodpasture syndrome was excluded because immunofluorescence analysis of the renal biopsy did not demonstrate the characteristic linear glomerular basement membrane immunoglobulin deposition. Likewise, although ANCA‐associated vasculitis was initially considered given the pulmonary–renal presentation and positive p‐ANCA, the absence of anti‐MPO and anti‐PR3 antibodies, together with the characteristic pulmonary histopathology and identification of a likely pathogenic COPA variant, supported COPA syndrome as the unifying diagnosis. The patient's positive p‐ANCA in the absence of anti‐MPO and anti‐PR3 antibodies is also noteworthy. Atypical ANCA positivity has been reported in patients with COPA syndrome and is thought to reflect the underlying immune dysregulation rather than primary ANCA‐associated vasculitis [7]. Therefore, ANCA positivity should be interpreted within the broader clinical, histopathologic and genetic context to avoid diagnostic misclassification.

Second, this case demonstrated severe and progressive pulmonary and renal involvement. The patient initially presented with haemoptysis and acute respiratory failure requiring mechanical ventilation, followed by progressive interstitial lung abnormalities and histopathologic evidence of follicular bronchiolitis. Renal disease was further supported by biopsy findings demonstrating focal proliferative and necrotising glomerulonephritis with early epithelial crescent formation, confirming active immune‐mediated renal involvement. The recurrence of renal deterioration after interruption of immunosuppressive therapy further emphasises the relapsing nature of COPA syndrome and the importance of sustained follow‐up and treatment adherence [1, 3].

The pulmonary histopathology in this case highlights the heterogeneous pattern of lung injury that can occur in COPA syndrome. The coexistence of follicular bronchiolitis, a cellular non‐fibrotic NSIP pattern, AFOP and both recent and remote diffuse alveolar haemorrhage reflects the underlying immune dysregulation rather than a single characteristic histopathologic pattern. Although none of these findings are specific in isolation, their combination, together with the clinical presentation and radiologic abnormalities, strongly supported the diagnosis of COPA syndrome following multidisciplinary evaluation and prompted confirmatory genetic testing.

The long‐term prognosis of COPA syndrome remains uncertain because of its rarity and marked clinical heterogeneity. Disease severity varies considerably, ranging from relatively stable disease to progressive multisystem involvement. Prognosis appears to depend primarily on the extent and progression of pulmonary and renal disease, particularly the development of progressive fibrotic interstitial lung disease, recurrent diffuse alveolar haemorrhage or chronic kidney dysfunction. Although life expectancy has not been well established, the principal causes of morbidity and mortality reported in patients with COPA syndrome include progressive respiratory failure, end‐stage kidney disease and complications related to immunosuppressive therapy, particularly severe infections. These observations underscore the importance of early diagnosis, close multidisciplinary follow‐up and careful monitoring to balance disease control with treatment‐related risks.

Overall, this case highlights the diagnostic challenges of COPA syndrome by illustrating an adult‐onset sporadic presentation with substantial diagnostic delay and a fatal complication during treatment, reinforcing the importance of early recognition and individualised multidisciplinary management.

Treatment of COPA syndrome focuses on controlling inflammation and preventing organ damage. Immunosuppressive therapies, such as corticosteroids and cyclophosphamide, have been commonly used, although their efficacy varies [1]. This patient initially responded to cyclophosphamide, achieving clinical stability for several years. However, disease recurrence necessitated further evaluation and consideration of alternative therapies.

Emerging treatments, such as Janus kinase (JAK) inhibitors, have shown promise in managing COPA syndrome by targeting the Type I interferon pathway. JAK inhibitors, including baricitinib and ruxolitinib, have demonstrated efficacy in reducing inflammation and improving symptoms in case reports and small studies [8]. These agents may be particularly beneficial for patients with severe or refractory disease.

This case also underscores the challenges associated with long‐term management of COPA syndrome. The fatal infectious complication observed during immunosuppressive treatment illustrates the delicate balance between controlling immune‐mediated disease activity and minimising treatment‐related adverse events. Patients receiving cyclophosphamide or other potent immunosuppressive agents may be particularly vulnerable to severe infections and therefore require careful multidisciplinary monitoring.

Because COPA syndrome is rare and phenotypically heterogeneous, diagnostic delays remain common, particularly when the clinical presentation overlaps with more prevalent autoimmune and interstitial lung diseases. Genetic testing plays a central role in establishing the diagnosis and should be considered in patients with unexplained interstitial lung disease accompanied by arthritis and nephropathy. Supportive care, including pulmonary rehabilitation and regular monitoring of pulmonary and renal function, remains essential to reduce complications such as progressive pulmonary fibrosis, respiratory failure and chronic kidney disease [9].

Further research is needed to establish standardised treatment strategies and evaluate the long‐term efficacy and safety of emerging therapies. Multicentre studies and international registries may improve understanding of the natural history of COPA syndrome.

In conclusion, this case highlights the importance of a multidisciplinary approach in diagnosing and managing rare autoimmune disorders like COPA syndrome. Genetic testing was instrumental in confirming the diagnosis and guiding treatment decisions. Early recognition, sustained treatment adherence and careful monitoring during immunosuppressive therapy are essential to prevent disease relapse and life‐threatening complications.

## Author Contributions

Flavia Castro Velasco Fernandes contributed to the conception of the study, patient evaluation, data collection and drafting of the manuscript. Lucas Velasco Magalhaes Fernandes and Matheus Rabahi contributed to the literature review, clinical data interpretation and manuscript preparation and submission. Pedro Paulo Teixeira e Silva Torres contributed to imaging interpretation and data analysis. Elisângela de Paula Silveira‐Lacerda contributed to clinical supervision and interpretation of findings. Matheus Rabahi supervised the study and performed critical revision of the manuscript for important intellectual content. Laís Rocha Lopes contributed by contacting the family for consent, reviewing and clinical evaluation of the case. Marise Amaral Rebouças Moreira contributed by pathological analysis and evaluation of the patient. And confirming clinical accuracy of the case presented.

## Funding

The authors have nothing to report.

## Consent

The authors declare that written informed consent was obtained for the publication of this manuscript and accompanying images using the consent form provided by the Journal.

## Conflicts of Interest

The authors declare no conflicts of interest.

## Data Availability

The data that support the findings of this study are available on request from the corresponding author. The data are not publicly available due to privacy or ethical restrictions.
